# Co-ingestion of cluster dextrin carbohydrate does not increase exogenous protein-derived amino acid release or myofibrillar protein synthesis following a whole-body resistance exercise in moderately trained younger males: a double-blinded randomized controlled crossover trial

**DOI:** 10.1007/s00394-021-02782-y

**Published:** 2022-02-19

**Authors:** Yusuke Nishimura, Mikkel Jensen, Jacob Bülow, Thomas Tagmose Thomsen, Takuma Arimitsu, Gerrit van Hall, Satoshi Fujita, Lars Holm

**Affiliations:** 1 School of Sport, Exercise and Rehabilitation Sciences, University of Birmingham, Edgbaston, Birmingham, B15 2TT UK; 2 Department of Sport and Health Science, Ritsumeikan University, Shiga, Japan; 3 Institute of Sports Medicine Copenhagen, Department of Orthopedic Surgery M, Bispebjerg Hospital, Copenhagen, Denmark; 4 Clinical Metabolomics Core Facility, Department of Clinical Biochemistry, Rigshospitalet, Copenhagen, Denmark; 5 Department of Biomedical Sciences, Faculty of Health and Medical Sciences, University of Copenhagen, Copenhagen, Denmark

**Keywords:** Resistance exercise, Muscle protein synthesis, Stable isotope tracer, Amino acids, Intrinsically labeled protein, mTORC1

## Abstract

**Purpose:**

This study investigates if co-ingestion of cluster dextrin (CDX) augments the appearance of intrinsically labeled meat protein hydrolysate-derived amino acid (D_5_-phenylalanine), Akt/mTORC1 signaling, and myofibrillar protein fractional synthetic rate **(**FSR).

**Methods:**

Ten moderately trained healthy males (age: 21.5 ± 2.1 years, body mass: 75.7 ± 7.6 kg, body mass index (BMI): 22.9 ± 2.1 kg/m^2^) were included for a double-blinded randomized controlled crossover trial. Either 75 g of CDX or glucose (GLC) was given in conjunction with meat protein hydrolysate (0.6 g protein * FFM^−1^) following a whole-body resistance exercise. A primed-continuous intravenous infusion of L-[^15^N]-phenylalanine with serial muscle biopsies and venous blood sampling was performed.

**Results:**

A time × group interaction effect was found for serum D_5_-phenylalanine enrichment (*P* < 0.01). Serum EAA and BCAA concentrations showed a main effect for group (*P* < 0.05). T_max_ serum BCAA was greater in CDX as compared to GLC (*P* < 0.05). However, iAUC of all serum parameters did not differ between CDX and GLC (*P* > 0.05). T_max_ serum EAA showed a trend towards a statistical significance favoring CDX over GLC. The phosphorylation of p70S6K^Thr389^, rpS6^Ser240/244^, ERK1/2^Thr202/Tyr204^ was greater in CDX compared to GLC (*P* < 0.05). However, postprandial myofibrillar FSR did not differ between CDX and GLC (*P* = 0.17).

**Conclusion:**

In moderately trained younger males, co-ingestion of CDX with meat protein hydrolysate does not augment the postprandial amino acid availability or myofibrillar FSR as compared to co-ingestion of GLC during the recovery from a whole-body resistance exercise despite an increased intramuscular signaling.

**Trial registration:**

ClinicalTrials.gov ID: NCT03303729 (registered on October 3, 2017).

## Introduction

Protein intake is essential for skeletal muscle protein adaptation to resistance exercise training [1]. Muscle protein synthesis is maximally stimulated when exercise is combined with protein ingestion [2–4]. Essential amino acids (EAA) have been shown to be potent in stimulating muscle protein synthesis [5, 6]. Mechanistically, leucine, one of the branched-chain amino acids (BCAA), is a potent stimulator of mammalian target of rapamycin complex 1 (mTORC1), which is a serine/threonine kinase regulating the translation and initiation [7–9]. Further, the availability of leucine and other essential amino acids [10, 11] and the achievement of higher peak aminoacidemia [12, 13] are associated with an increase in the rate of muscle protein synthesis, suggesting the importance of quick absorption and rise in essential amino acid availability in the circulation to induce muscle protein synthesis [12, 14].

Carbohydrate is often added to protein supplementations for various reasons, such as increasing energy intake, providing readily available substrate for energy metabolism, and improving taste. However, the uptake rate of exogenous amino acids and their availability in circulation is attenuated when consumed with carbohydrate [15–17] or a mixed macronutrients meal [18, 19]. This is due to a decrease in the rate of digestion and absorption [16] and an increase in the retention of amino acids in the portal drained viscera [20]. In addition, amino acid availability in the circulation is reduced by suppression of protein breakdown [21, 22]. Although plasma insulin concentration was greater when protein was ingested with carbohydrate [15–17] or a mixed meal [18], muscle protein synthesis was not augmented as compared to protein intake alone [15–17]. A systematic review concluded that a systemic administration of insulin does not have a stimulatory or inhibitory effect for muscle protein synthesis and that amino acid availability dictates muscle protein synthesis in healthy younger individuals [23]. Thus, the current evidence does not provide the interactive effect of carbohydrate co-ingestion with protein to augment muscle protein synthesis. However, if the addition of carbohydrate delays the availability of amino acids in the circulation, it could be speculated that carbohydrate would result in a delayed increase in muscle FSR, as observed in slower digestible proteins [13].

Cluster dextrin (CDX) is a branched carbohydrate produced from waxy maize starch by the cyclization of a branching enzyme [24]. CDX is highly soluble in water, has low viscosity, and has a relatively low tendency for retrogradation [25] compared to commercial dextrin [26]. CDX has also been shown to increase the rate of gastric emptying compared to glucose (GLC) and standard dextrin due to a lower osmotic pressure [27]. Accordingly, the rapid gastric emptying of CDX might alleviate the lower amino acid availability when co-ingested with protein as compared to GLC. Thus, a measurement of exogenous protein-derived amino acid availability (i.e., a downstream measurement of digestion and absorption) is required to determine whether co-ingestion of CDX increases amino acid availability for the periphery.

Therefore, we hypothesized that the appearance of amino acids from orally ingested meat protein hydrolysate into the circulation would be faster when it is co-ingested with CDX than GLC. Accordingly, we further hypothesized that the ingestion of the meat protein hydrolysate with CDX would result in a greater Akt/mTORC1 signaling response and myofibrillar FSR following an acute bout of whole-body resistance exercise as compared to GLC in moderately trained younger males.

## Methods

### Ethical approval

This study was approved by the Ethics Committee of the Capital Region (H-17017363) and adhered to the Helsinki II declaration. Before inclusion, each participant was informed of the purpose of the study, experimental procedures, and potential risks prior to obtaining written informed consent. This trial was registered at clinicaltrails.gov as NCT03303729.

### Subjects

Ten moderately trained healthy males (21.5 ± 2.1 years, 22.9 ± 2.1 kg/m^2^; values are mean ± SD) volunteered to participate in a double-blinded, randomized controlled crossover study. Inclusion criteria were as follows: healthy men who conduct structured whole-body resistance training between 1 and 3 times (1–3 h) per week on average over the last 3 months. Exclusion criteria were as follows: subjects younger than 18 years or above 30 years of age, BMI > 30, smoking, active cancer, renal diseases, diabetes mellitus, vegetarian, physical inactivity (i.e., no systematized exercise), and perform systematized resistance exercise more than 3 times per week. Baseline subject characteristics are presented in Table 1.

**Table 1:** Baseline subject characteristics

Age, y	21.5 ± 2.1
Height, m	1.82 ± 0.54
Body mass, kg	75.7 ± 7.6
FFM, kg	60.4 ± 5.4
BMI, kg/m^2^	22.9 ± 2.1
1-RM leg press, kg	283.0 ± 50.6
1-RM leg extension, kg	116.5 ± 12.1
10-RM shoulder press, kg	50.6 ± 4.5
10-RM pulldown, kg	56.5 ± 9.0

All values are presented as means ± SD. *n* = 10

*FFM* fat free mass, *BMI* body mass index

### Study overview

**Fig. 1:**
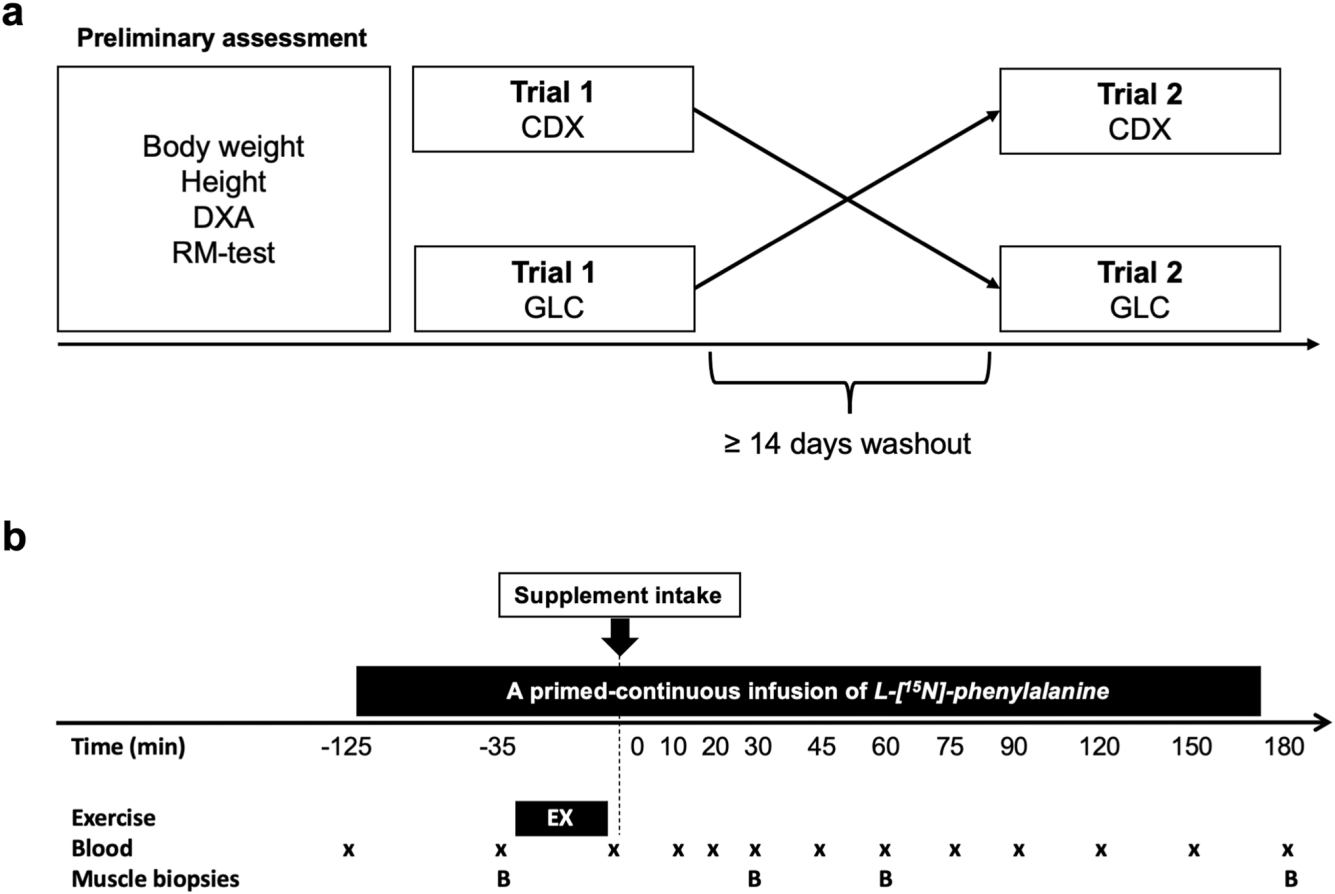
Schematic overview of crossover study design (**a**) and experimental protocol (**b**). *DXA* dual X-ray absorptiometry; *RM* repetition maximum, *EX* exercise, *B* muscle biopsy, *GLC* glucose, CDX cluster dextrin

**Fig. 2:**
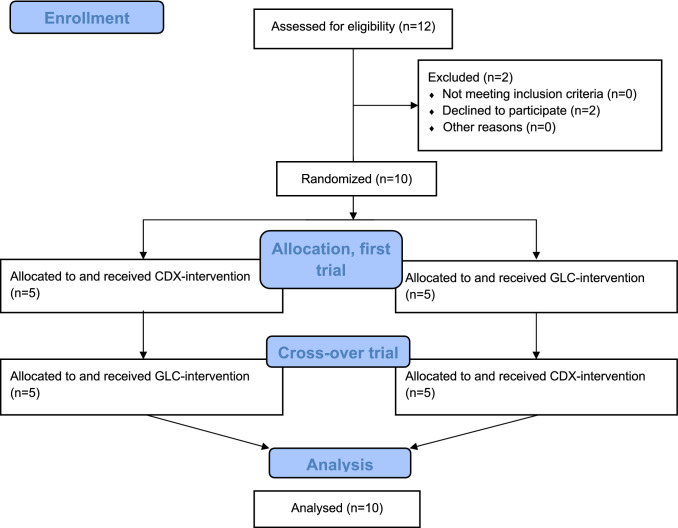
A CONSORT flowchart diagram

This was a double-blinded randomized controlled crossover trial conducted at The Institute of Sports Medicine Copenhagen (ISMC), Bispebjerg Hospital. The overall timeline of the study is shown in Fig. 1a, and a CONSORT flowchart diagram is displayed in Fig. 2. Briefly, at least 2 weeks prior to the first trial, subjects underwent preliminary assessments. The two experimental trials were separated by at least 2 weeks to minimize any interaction on the second trial from the previous trial. On both trial days, the participants arrived in an overnight fasted state at 08:00 h at The Institute of Sports Medicine Copenhagen (ISMC), Bispebjerg Hospital. Personnel with no direct involvement in the experiment rolled dice and created a scheme where the order of the interventions (CDX or GLC) was in code for each subject. On the day of an experiment, the personnel prepared the designated intervention and handed it over to the investigator (MJ). Thus, the allocation of interventions was concealed from the participants and the study investigator until the completion of data analysis. In each experimental visit, muscle biopsies and blood samples were obtained during a primed-continuous stable isotope amino acid infusion (^15^N-phenylalanine) to determine amino acid availability from orally ingested intrinsically labelled meat protein hydrolysate (D_5_-phenylalanine), myofibrillar muscle protein synthesis, and intracellular signaling in response to a whole-body resistance exercise and the intake of meat protein hydrolysate with either GLC or CDX.

### Preliminary assessments

#### Body mass and height

Height was measured in the upright position without shoes against a wall and body weight was measured on a digital scale (Seca 719, Seca gmbh & co., Hamburg, Germany) in light clothing.

#### Body composition

Dual X-ray absorptiometry (DXA) was performed to determine the whole-body fat free mass (FFM) by using the enCORE v.16 software (Lunar iDXA; GE Medical Systems, Pewaukee, WI, USA) after having emptied their bladder. DXA scans were performed after at least 12 h overnight fast.

#### Strength tests

After the scanning, strength was assessed by leg press, knee extension (Super Executive Line, TechnoGym, Gambettola, Cesana, Italy), shoulder press (TR Equipment model 9025, Tranås, Sweden) and shoulder pull/pull-down (Lat Mach, TechnoGym, Gambettola, Cesana, Italy) strength exercise machines. The strength tests were 1 repetition maximum (RM) and 10RM for leg exercises and upper body exercises, respectively. The 10RM test for the upper body exercises was chosen to minimize any risk of injury by unaccustomed exercises during testing.

### Experimental protocol

The experimental protocol is shown in Fig. 1b. The overall experimental protocol consisted of a primed (4.0 µmol * kg FFM^−1^) continuous (3.8 µmol * kg FFM^−1^ * hour^−1^) infusion of L-[^15^N]-phenylalanine, which was applied over the course of the experimental trial. The participants rested in the supine position for the remainder of the trial day, only interrupted by the training program and toilet visits. Two antecubital catheters were inserted into each arm of the participant, one for the infusion of a stable isotope amino acid tracer (^15^N-phenylalanine) and the other for serial blood sampling. A background blood sample was drawn after which the primed continuous infusion was started. The subject rested for approximately 1.5 h after which the first biopsy was taken, and a blood sample was drawn. The leg and site of the biopsies were randomized using dice. Hereafter, the participant walked a small distance of 100 m to the training facility where they conducted the resistance exercise program under full guidance and supervision (see Resistance exercise protocol). Upon completion, the participant walked back to the trial room where a post-exercise blood sample was drawn. Hereafter, an independent research assistant prepared the drink containing the intrinsically labeled (D_5_-phenylalanine) meat protein hydrolysate (0.6 g protein * FFM^−1^) mixed with either 75 g of GLC or CDX in 350 mL of cold tap water. The drink was consumed in less than 5 min by the participant. Furthermore, the post-exercise drink was enriched with 5% ^15^N-phenylalanine to minimize any fluctuations in the serum enrichment after consumption of the protein-rich drink (unlabeled phenylalanine). The amount of meat protein hydrolysate was decided to provide sufficient amino acids following a whole-body resistance exercise. A previous study showed that a higher protein intake is required when a whole-body resistance exercise is performed as compared to a unilateral leg resistance exercise [28]. Nutritional composition of meat protein hydrolysate, CDX, GLC is presented in Table 2. Once the drink was consumed, blood was sampled in a vacutainer coated with Z serum clot activator (Vacuette tube, Greiner Bio-One GhmB, Austria) at 10, 20, 30, 45, 60, 75, 90, 120, 150, and 180 min following the post-exercise drink. Muscle biopsies were taken from the vastus lateralis at 30, 60, and 180 min post drink under local anesthesia (1% lidocaine) using the Bergström technique [29]. Muscle samples were rinsed in ice-cold saline (9 mg/ml) and freed from any visible blood and connective tissues before being snap frozen in liquid nitrogen and stored at -80 °C for future analysis. After the last biopsy, the infusion was stopped, catheters were removed, and the participant was given a sandwich before being sent home.

**Table 2:** Nutritional composition of meat protein hydrolysate, cluster dextrin (CDX), and glucose (GLC)

	Meat protein hydrolysate (0.6 g protein/ FFM kg)^1^	CDX	GLC
Total served weight, g	300	75	75
Energy, kcal	162	300	300
Protein, g	36	–	–
Protein, kcal	144	–	–
Fat, g	1.8	–	–
Fat, kcal	16.2	–	–
Carbohydrate, g	0	75	75
Carbohydrate, kcal	0	300	300
Alanine, g	2.04	–	–
Arginine, g	2.06	–	–
Aspartic acid, g	3.27	–	–
Cysteine, g	0.28	–	–
Glutamic acid, g	5.46	–	–
Glycine, g	1.46	–	–
Histidine, g	1.36	–	–
Isoleucine, g	1.61	–	–
Leucine, g	2.86	–	–
Lysine, g	3.21	–	–
Methionine, g	0.80	–	–
Phenylalanine, g	1.54	–	–
Proline, g	1.27	–	–
Serine, g	1.37	–	–
Threonine, g	1.61	–	–
Tryptophan, g	0.42	–	–
Tyrosine, g	1.17	–	–
Valine, g	1.74	–	–
Total essential amino acids, g	15.16	–	–
Total amino acids, g	33.55	–	–
D_5_-phenylalanine enrichment^2^, MPE ± SD	0.73 ± 0.01	–	–

*CDX* cluster dextrin, *GLC* glucose, *MPE* mole percent excess, *SD* standard deviation

^1^For an individual with 60 fat free mass (FFM) kg

^2^Value was reported by Reitelseder et al. [18]

#### Resistance exercise protocol

The resistance exercise session consisted of four different exercises for both the lower and upper body and was the same as those used in the preliminary assessment: leg press, knee extension, shoulder press and shoulder pull/pull down. The subjects completed 3 sets of 8 repetitions at 70% of 1RM in each of the two leg exercises and 3 sets of 10 reps at 10RM in the two upper body exercises. Three sets of each exercise were completed before conducting the next exercise. Rest periods of 2 min were allowed between sets and exercises.

#### Meat protein hydrolysate

The meat, from which the hydrolysate has been produced, had been intrinsically labeled with ring-D_5_-phenylalanine by infusing Holstein cows with the ring-D_5_-phenylalanine tracer for 72 h before slaughter [18]. The meat protein hydrolysate is quickly absorbed and induces an immediate and high availability of amino acids in the circulation after a bolus intake [18]. This allows to directly measure the availability of the orally consumed meat protein hydrolysate-derived amino acids in the circulation.

### Blood analysis

Blood was sampled with vials coated with Z serum clot activator and left at room temperature for 30 min before being centrifuged (3,970 x g, 10 min, 4 °C) in an Eppendorf 5810R (Eppendorf AG, Hamburg, Germany) to obtain serum. Serum samples were stored at -80 °C for further analysis.

Serum amino acid concentrations were determined as described in detail elsewhere [30]. We used 100 µL of the serum, which was added internal standards for all amino acids and were acidified with the addition of 120 µL of 50% acetic acid before being poured over columns (Medium HDPE Open tip column CC07, Intertech Medical Inc., Denver, CO) containing acidified cation exchange resin (Dowex AG 50 W-X8 resin 100–200 mesh, BioRad, Copenhagen, Denmark). Purified amino acids were converted to their phenylthiocarbamyl (PTC) derivatives, by adding a coupling buffer (methanol: Milli-Q® water:triethylamin (2:2:1, %, v/v)), drying at 70 °C under a flow of N_2_, adding the derivatization solution (triethylamin: Milli-Q® water:PITC:methanol (1:1:1:7, %, v/v)). Then, the sample was vortexed and incubated at room temperature for 30 min. Hereafter, the solution was dried at 70 °C under a flow of N_2_ and acetonitrile, methanol, and Milli-Q® purified water (44:10:46, %, v/v) with 0.1 M ammonium acetate was added. The isotope ratios of D_5_- and ^15^N-phenylalanine and a full amino acid concentration profile were determined on a liquid chromatography–tandem mass spectrometer (LC–MS/MS; triple stage quadrupole mass spectrometer, TSQ Vantage, Thermo Fischer Scientific, San Jose, CA, USA).

Serum insulin concentrations were measured using a high-sensitivity human insulin enzyme-linked immunosorbent assay (ELISA) kit (DRG Instrument GmbH) at the time points − 35, 0, 10, 20, 30, 45, 60, 90, 120, and 180 min.

### Muscle tissue analyses

#### Intramuscular amino acid concentration

From 10 mg of wet weight muscle, BCAA concentrations were determined using the same protocol as for serum amino acids as described above [30]. Briefly, the frozen muscle specimens were homogenized in 1 ml of 6% perchloric acid with an added internal standard for the determination of BCAA concentrations. The samples were spun down and the supernatant containing the tissue free amino acids was extracted. The samples were then poured over acidified cation exchange columns with resin (AG 50 W-X8 resin, Bio-Rad laboratories, Hercules, Ca, USA). The amino acids were eluted with 2 × 2 ml 4 M NH_4_OH and derivatized into their phenylthiocabamyl (PTC) derivative. Derivatized samples were loaded and analyzed on LC–MS/MS (Thermo Fischer Scientific, San Jose, CA, USA). Each intramuscular BCAA concentration was normalized to the wet weight of muscle used to prepare for the analysis. The intramuscular water fraction was set as 0.77 of the muscle wet weight.

#### Myofibrillar protein bound tracer enrichments

From 20 mg of wet weight muscle, the abundance of myofibrillar protein bound ^15^N-phenylalanine was measured according to our lab’s standard protocol. Briefly, the frozen muscle specimen was homogenized in 1 mL of buffer (Tris 0.02 M [pH 7.4], NaCl 0.15 M, EDTA 2 mM, EGTA 2 mM, TritonX-100 0.5%, sucrose 0.25 M) for 4 × 45 s, speed 5.5 (FastPrep 120A-230; Thermo Savant, Holbrook, NY, USA) and left at 5 °C for 3 h. Hereafter, samples were centrifuged at 800 g for 20 min at 5 °C. The supernatant was discarded, and 1 mL of homogenization buffer was added, homogenized for 45 s at speed 5.5, incubated at 5 °C for 30 min, and centrifuged. The supernatant was discarded, and 1.5 mL buffer (KCl 0.7 M, pyrophosphate (Na_4_P_2_O_7_) 0.1 M) was added to the pellet, vortexed and left overnight at 5 °C. The day after, the samples were spun at 1,600 x g for 20 min at 5 °C and the supernatant transferred to glass vials suitable for hydrolysis and added 2.3 × vol ethanol 99%, vortexed and left for 2 h at 5 °C, and subsequently spun at 1,600 x g for 20 min. The supernatant was then discarded, and 1 mL of 70% ethanol was added to the pellet, after which the solvent was vortexed and centrifuged at 1,600 x g for 20 min. The supernatant was discarded and 1 mL of 1 M HCl and 1 mL of resin slurry was added to the pellet and left overnight at 110 °C. The solvent was diluted with water and the amino acids were purified over cation exchange resin columns. The purified amino acids were derivatized as their N-acetyl-propyl (NAP) derivate and analyzed on gas chromatograph-combustion-isotope ratio mass spectrometer by following standard procedure described thoroughly by Bornø et al. [31].

#### Intracellular signaling

Western blotting was performed as reported previously with a slight modification [32]. Briefly, approximately ~ 30 mg of frozen muscle tissue samples was homogenized in tenfold volumes of RIPA buffer (Cell Signaling Technology, Danvers, MA, USA) supplemented with protease and phosphatase inhibitor cocktail (Roche Life Science, Indianapolis, IN, USA) per 10 mL of homogenization buffer. The resulting homogenates were centrifuged at 14,000 x g for 10 min at 4 °C. The supernatant was transferred to a new vial and total protein concentrations were determined by the Protein Assay Rapid kit (WAKO, Osaka, Japan). The samples were standardized to 2 μg protein per 1 μL by dilution with 3 × SDS sample buffer containing 15% β-mercaptoethanol, 6% SDS, 187.5 mM Tris–HCl (pH 6.8), 15% sucrose, and 0.015% bromophenol blue and boiled at 95 °C for 5 min. An equal amount of protein (10 μg) was loaded into each lane and the samples were separated by electrophoresis on a 10 or 15% SDS–polyacrylamide gel for 45 min at 250 V. Following electrophoresis, proteins were transferred to a polyvinylidene fluoride (PVDF) membrane for 1 h at 20 V via a semi-dry transfer. Membranes were subsequently blocked in 5% milk for 1 h at room temperature. After blocking, membranes were washed 3 times for 5 min in Tris-buffered saline with 0.1% Tween (TBST) before being incubated overnight at 4 °C in primary antibody against phospho-70 kDa S6 protein kinase (p70S6K) Thr389 (#9234), total p70S6K (#2708), phospho-eukaryotic initiation factor 4E-binding protein (4E-BP1) Thr37/46 (#9459), total 4E-BP1 (#9452), phospho-protein kinase B (Akt) Ser473 (#9271), total Akt (#2920), phospho-AMPKα Thr172 (#2535), total AMPKα (#2793), phospho-ribosomal protein S6 (rps6) Ser240/244 (#2215), total rps6 (#2217), phospho–eukaryotic elongation factor 2 (eEF2) Thr56 (#2331), total eEF2 (#2332) each purchased from Cell Signaling Technology (Danvers, MA, USA). Membranes were then washed again 3 times for 5 min in TBST and incubated for 1 h in their respective secondary antibody at room temperature and washed again 3 times for 5 min in TBST. Chemiluminescence (Luminata 200 Forte Western HRP Substrate; Merck Millipore, Temecula, CA, USA) was applied to each blot. Images were developed using an ImageQuant LAS 4000 (GE Healthcare, Amersham, UK). Band intensities were quantified using Image Studio Lite (Li-Cor, Lincoln, Nebraska, USA). Phosphorylation levels were determined by the expression of phosphorylated protein divided by the expression of non-phosphorylated total protein. The membranes were stained with Ponceau-S to verify equal loading and used as the normalization control.

### Calculations

FSR was calculated using the precursor-product method [33]:

1 $$\mathrm{FSR}=\frac{{\mathrm{E}}_{2}-{E}_{1}}{{\mathrm{\acute{e} }}_{t1-t2}*{t}_{1-2}} * 100,$$

where E is the protein bound enrichment, é is precursor enrichment between two samples estimated from venous serum samples and t is the time between two samples. The FSR will be calculated from 30 to 180 min post exercise.

### Statistical analysis

To compare the effect of time within each of the two trials and the two dependent trials (GLC versus CDX), we applied a 2-factor [time × group (GLC compared with CDX)] repeated measures ANOVA when no missing data appeared and a mixed-effects model when data points were missing (insulin concentration measures; one data point was missing (Subject#1, CDX trial, time point 180 min). Turkey’s multiple comparisons test was used as a post hoc test to identify the individual differences when there was a time × group interaction effect, a main effect of time, or a main effect of group. Paired student two-tailed *t*-test was used to compare postprandial (0.5–3 h) FSR between CDX and GLC trials, incremental area under the curve (iAUC), and time to reach maximum concentration (T_max_) for serum D_5_-phenylalanine enrichments and serum phenylalanine, EAA, BCAA, and insulin concentrations. iAUC is the definite integral of a curve that depicts the serum parameters as a function of time during the postprandial period using the value for the parameter at time point zero as the baseline value. iAUC was computed using the trapezoid rule. A straight line is connected between adjunct points, and the beneath area was calculated as ΔX*([(Y1 + Y2)/2]-Baseline]. This was repeated for each region, and the sum of the areas was defined as iAUC. T_max_ was obtained from the concentration–time data, where the time of the highest concentration of serum parameters was observed during the postprandial period. A priori power analysis was performed for a matched paired *t*-test (two tails) with an α error probability = 0.05, power (1-β error probability) = 0.8, and Cohen’s effect size dz = 1.0 using G^∗^Power version 3.1 analysis software (Heinrich Hein University). Cohen’s effect size dz = 1.0 was calculated based on the least detectable difference of 0.01%/h FSR between groups and the within subject standard deviation of 0.01%/h. This produced a minimum sample size of n = 10. Data are expressed as means ± SD or SEM. An alpha level of 0.05 was used to determine statistical significance. All statistical analysis was performed using GraphPad Prism version 8.4.3 for Mac (GraphPad Software, La Jolla California USA).

## Results

### Participants

Baseline subject characteristics are shown in Table 1. Figure 2 shows a CONSORT flow diagram describing the progress from recruitment through completion of the study.

### Phenylalanine amino acid tracer enrichment

**Fig. 3:**
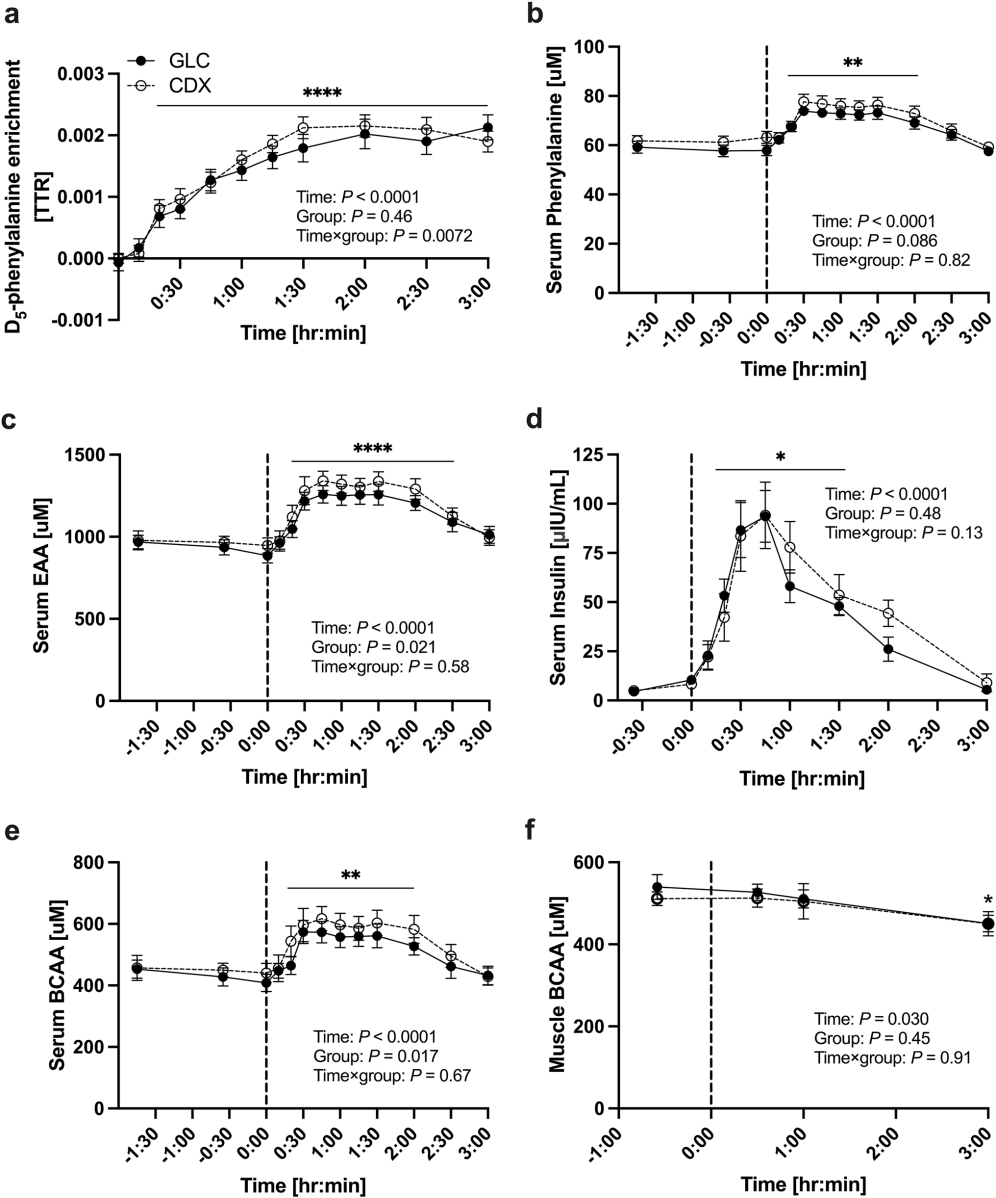
The time course of serum D_5_-phenylalanine enrichment (**a**), phenylalanine (**b**), EAA (**c**) insulin (**d**) BCAA (**e**) muscle BCAA (**f**) concentrations. The vertical dot line on each graph (at *t* = 0) indicates the transition from postabsorptive to postprandial conditions via the ingestion of meat protein hydrolysate (0.6 g protein * FFM^−1^) with either 75 g of GLC (*n* = 10) or CDX (*n* = 10) following a whole-body resistance exercise. Data were analyzed with the use of a 2-factor [time × group (GLC compared with CDX)] ANOVA with Turkey’s multiple comparisons test to locate individual differences. Values are means ± SEM. Significance was set at *P* < 0.05. There was a main effect of time for serum D_5_-phenylalanine enrichment, phenylalanine, EAA, BCAA, insulin concentrations and (*P* < 0.0001) and muscle BCAA (*P* < 0.05). There was a main effect of group (GLC compared with CDX) for serum EAA and BCAA concentrations (*P* < 0.05). There was a time × group interaction effect for D_5_-phenylalanine enrichment (*P* < 0.05). *, **, **** denotes significant difference from basal (*P* < 0.05, *P* < 0.01, *P* < 0.0001, respectively). *TTR* tracer to tracee ratio, *GLC* glucose, *CDX* cluster dextrin, *BCAA* branched-chain amino acids

**Fig. 4:**
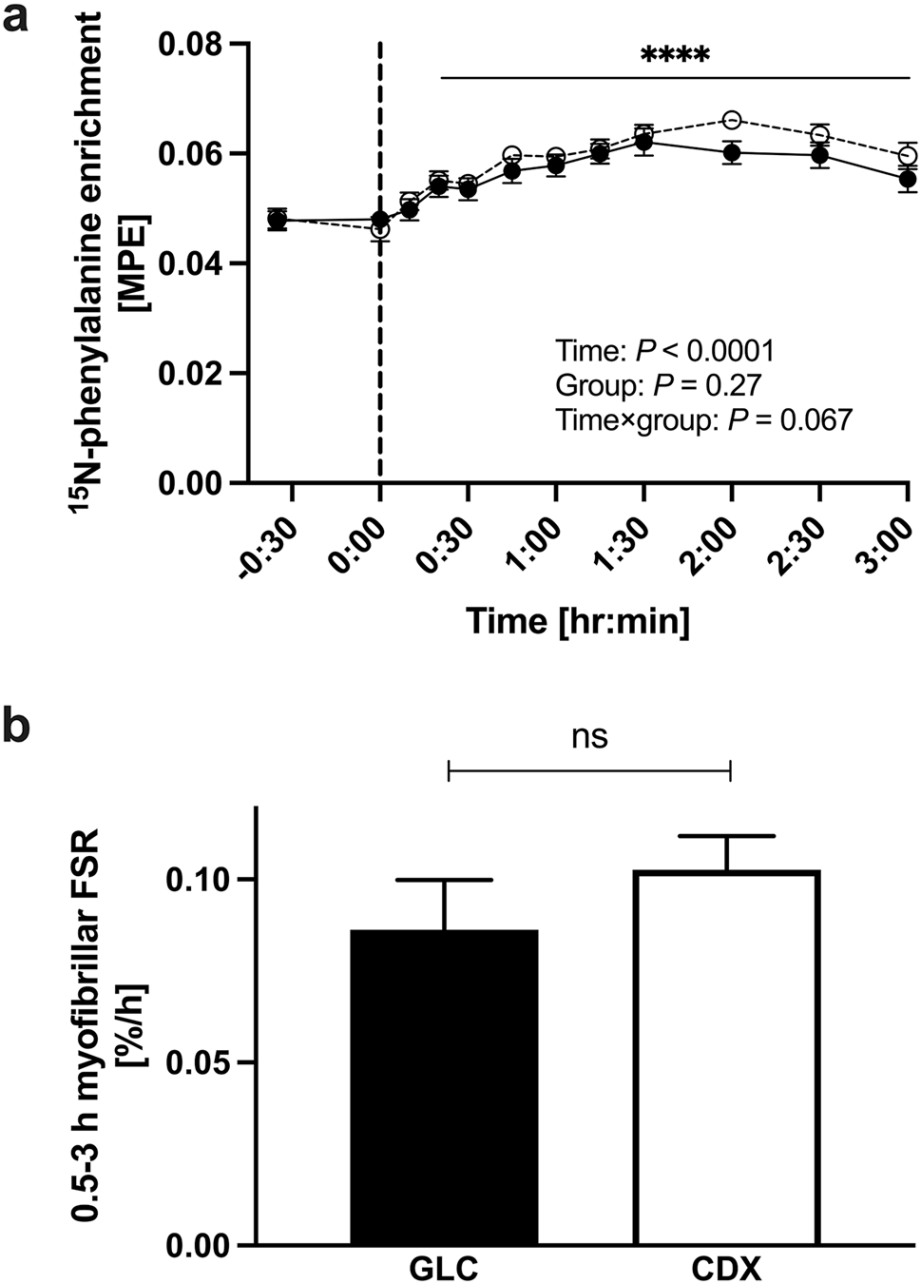
The time course of serum ^15^N-phenylalanine enrichment (**a**) and myofibrillar FSR over a 2.5-h postprandial period (**b**). The vertical dot line on each graph (at *t* = 0) indicates the transition from postabsorptive to postprandial conditions via the ingestion of meat protein hydrolysate (0.6 g protein * FFM^−1^) with either 75 g of GLC (*n* = 10) or CDX (*n* = 10) following a whole-body resistance exercise. Serum ^15^N-phenylalanine enrichment was analyzed with the use of a 2-factor [time × group (GLC compared with CDX)] ANOVA with Turkey’s multiple comparisons test to locate individual differences. Values are means ± SEM. Significance was set at *P* < 0.05. There was a main effect of time for serum ^15^N-phenylalanine enrichment (*P* < 0.0001). Myofibrillar FSR was analyzed with the use of a paired *t*-test (two-tailed). *n* = 10/group. Values are means ± SEM. Significance was set at *P* < 0.05. Analysis revealed no statistical difference between GLC and CDX (*P* = 0.17). *MPE* mole percent excess, *FSR* fractional synthesis rate, *GLC* glucose, *CDX* cluster dextrin

Serum D_5_-phenylalanine (Fig. 3a) enrichment originated from intrinsically labeled meat protein hydrolysate was increased following the ingestion of post-exercise drink at *t* = 0 (a main effect of time, *P* < 0.0001) between 20 and 180 min (*P* < 0.0001). There was no main effect of group for serum D_5_-phenylalanine enrichment (*P* = 0.46). There was a time × group interaction effect (*P* = 0.0072). The enrichment of the infused tracer, L-[ring-^15^N]-phenylalanine (Fig. 4a), was elevated above basal value (*t* = 0) following the tracer infusion 20–180 min (a main effect of time, *P* < 0.0001). There was no main effect of group (*P* = 0.27) and time × group interaction effect (*P* = 0.067).

### Serum amino acid concentrations

Serum phenylalanine concentrations (Fig. 3b) were increased compared to basal value (*t* = 0) from 20 to 120 min (*P* < 0.01) following the intake of post-exercise drink (a main effect of time, *P* < 0.0001). There was no main effect of group for serum phenylalanine (*P* = 0.086). Serum EAA concentrations (Fig. 3c) were increased compared to basal value (*t* = 0) from 20 to 150 min (*P* < 0.0001) following the intake of post-exercise drink (a main effect of time, *P* < 0.0001). There was a main effect of group for serum EAA (*P* = 0.021). Likewise, serum BCAA concentrations (Fig. 3e) were increased above basal value (*t* = 0) between 20 and 120 min (*P* < 0.01) with an overall time effect (*P* < 0.0001). There was a main effect of group for serum BCAA concentrations (*P* = 0.02). In contrast, there was a main effect of time (*P* < 0.05) for muscle BCAA concentrations (Fig. 3f), and it was decreased at 180 min (*P* < 0.05) following the intake of post-exercise drink compared to basal value (t = − 35 min). There was no main effect of group for muscle BCAA (*P* = 0.45). There was no time × group interaction effect for serum EAA (*P* = 0.58), phenylalanine (*P* = 0.82), BCAA concentrations (*P* = 0.67), and muscle BCAA concentrations (*P* = 0.91).

### Serum insulin concentrations

Serum insulin concentrations (Fig. 3d) were increased above basal value (*t* = 0) between 20 and 90 min (*P* < 0.05) following the intake of post-exercise drink (an overall time effect, *P* < 0.0001). There was no main effect of group (GLC compared with CDX) for serum insulin concentrations (*P* = 0.48). There was no time × group interaction effect for insulin concentrations (*P* = 0.13).

### iAUC and T_max_ in serum parameters

iAUC and T_max_ serum D_5_-phenylalanine, phenylalanine, EAA, BCAA, and insulin are displayed in Table 3. T_max_ serum BCAA was higher in CDX compared to GLC (*P* < 0.05). T_max_ serum EAA showed a trend towards statistical significance favoring CDX over GLC (*P* = 0.051).

**Table 3:** iAUC and T_max_ serum D_5_-phenylalanine, phenylalanine, EAA, BCAA, and insulin in response to meat protein hydrolysate intake with either GLC or CDX

	GLC	CDX	*P* value
D_5_-phenylalanine enrichment			
iAUC, TTR min	0.0048 ± 0.00033	0.0050 ± 0.00029	0.46
T_max_, min	133.5 ± 13.5	120 ± 11.0	0.51
Phenylalanine			
iAUC, μM min	32.1 ± 2.3	27.0 ± 2.9	0.099
T_max_, min	75.0 ± 7.7	69.0 ± 11.7	0.68
EAA			
iAUC, μM min	825.2 ± 54.6	810.1 ± 64.2	0.84
T_max_, min	84.0 ± 7.8	64.5 ± 9.8	0.051
BCAA			
iAUC, μM min	335.8 ± 24.7	342.4 ± 37.4	0.87
T_max_, min	70.5 ± 11.0	47.0 ± 8.0	0.049*
Insulin			
iAUC, μIU/mL min	94.6 ± 10.8	119.0 ± 17.4	0.098
T_max_, min	51.0 ± 8.4	39.5 ± 3.7	0.29

All values are presented as means ± SEM. *n* = 10. Paired student two-tailed *t* test was used to compare between GLC and CDX. Significance was set at *P* < 0.05

*iAUC* incremental area under the curve, *T*_*max*_ time to reach maximum concentration, *EAA* essential amino acids, *BCAA* branched-chain amino acids, *CDX* cluster dextrin, *GLC* glucose, *TTR* tracer to tracee ratio, *SEM* standard error of mean, *IU* international unit

*Significant difference between groups (*P* < 0.05)

### Myofibrillar protein fractional synthesis rate

Postprandial myofibrillar FSR was calculated using the average serum L-[ring-^15^N]-phenylalanine enrichments as the precursor pool (Fig. 4b). Myofibrillar protein FSR between 30 and 180 min was not different between GLC and CDX (0.0862 ± 0.0137 and 0.1026 ± 0.0093%•h^−1^, respectively, *P* = 0.17).

### Intracellular signaling

**Fig. 5:**
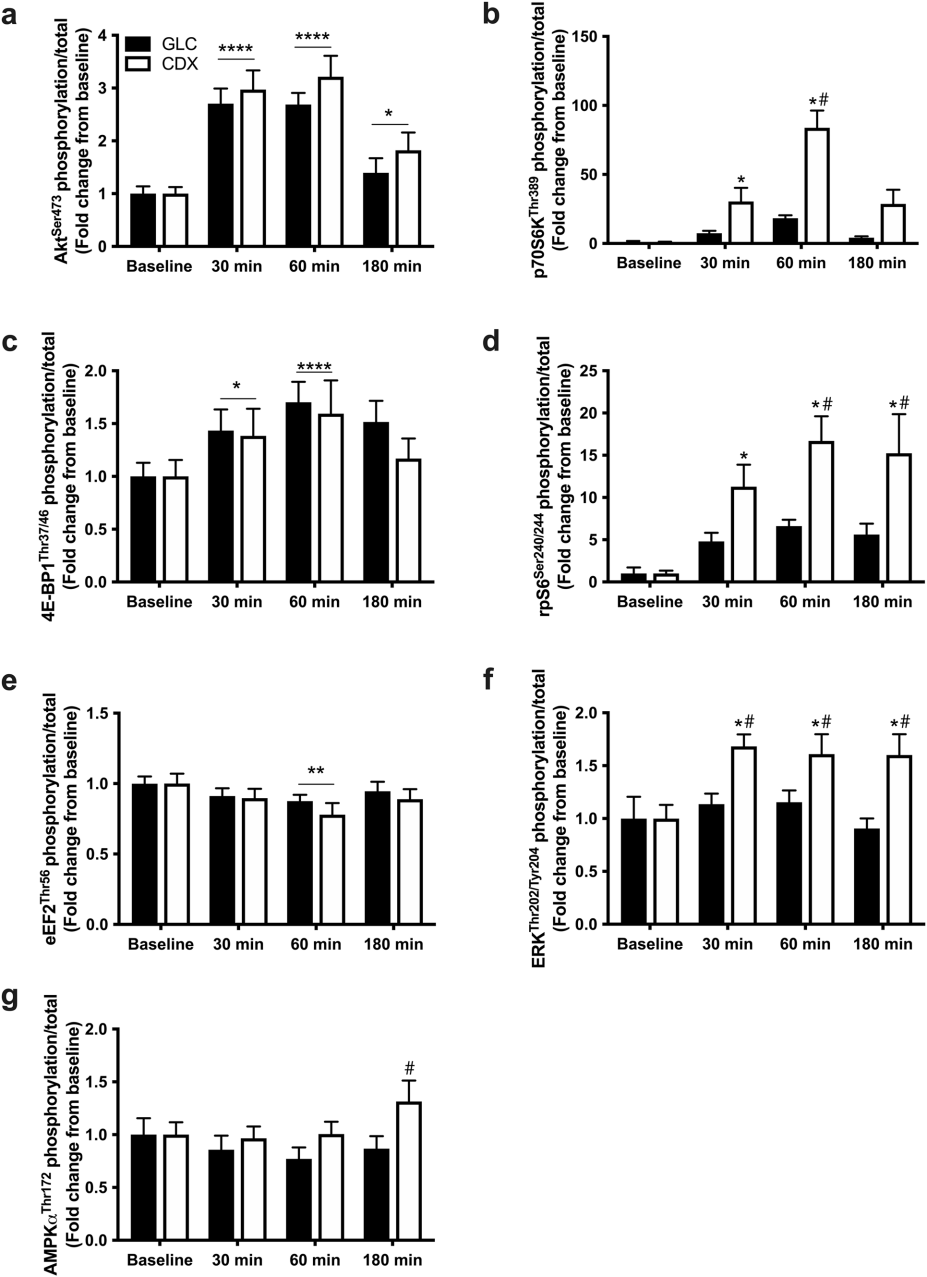
The phosphorylation of Akt^Ser473^ (**a**), p70S6K^Thr389^ (**b**), 4E-BP1^Thr37/46^ (**c**), rpS6^Ser240/244^ (**d**), eEF2^Thr56^ (**e**), ERK1/2^Thr202/Tyr204^ (**f**), AMPKα^Thr172^ (**g**) at 30, 60, 180 min after the ingestion of meat protein hydrolysate (0.6 g protein * FFM^−1^) with either GLC (*n* = 10) or CDX (*n* = 10). Data were analyzed with the use of a 2-factor [time × group (GLC compared with CDX)] ANOVA with Turkey’s multiple comparisons test to locate individual differences. The data were expressed relative to baseline. Values are means ± SEM. There was a main effect of time for Akt^Ser473^, p70S6K^Thr389^, rpS6^Ser240/244^, 4E-BP1^Thr37/46^, eEF2^Thr56^, ERK1/2^Thr202/ Tyr204^, and AMPKα^Thr172^ (*P* < 0.05). There was a main effect of group (GLC compared with CDX) for Akt^Ser473^ p70S6K^Thr389^, rpS6^Ser240/244^, ERK1/2^Thr202/ Tyr204^ (*P* < 0.05). There was a time × group interaction effect for p70S6K^Thr389^, rpS6^Ser240/244^, ERK1/2^Thr202/ Tyr204^, AMPKα^Thr172^ (*P* < 0.05). Significance was set at *P* < 0.05. *, **, **** denotes significant difference from baseline in respective group (*P* < 0.05, *P* < 0.01, *P* < 0.0001). ^#^ indicates significant difference between CDX and GLC at the same time point (*P* < 0.05). *GLC* glucose, *CDX* cluster dextrin

**Fig. 6:**
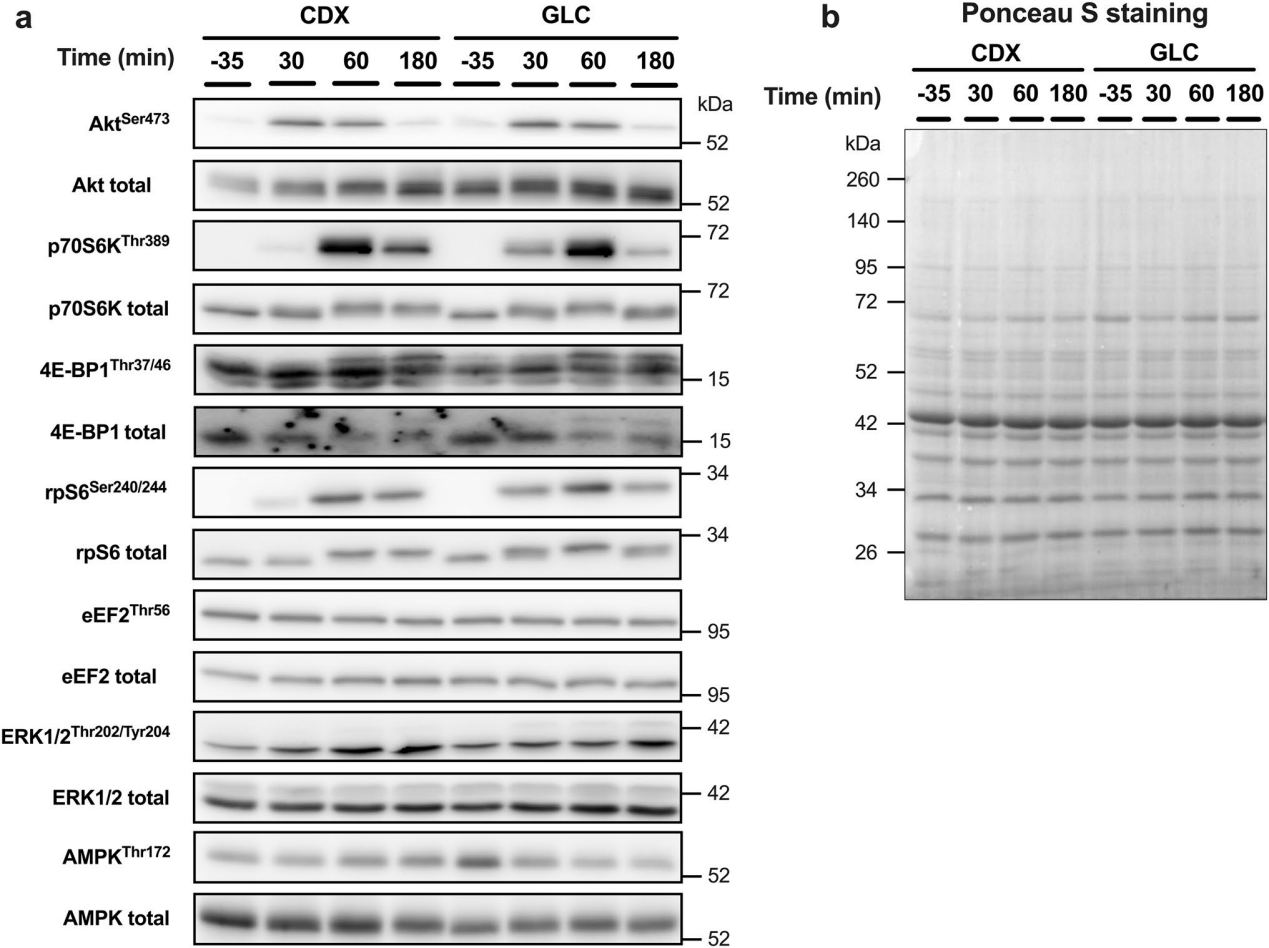
Representative western blot images for intracellular signaling (**a**) and Ponceau-S (**b**). *CDX* cluster dextrin, *GLC* glucose

The time-dependent changes of intracellular signaling are displayed in Fig. 5 and representative western blot images are shown in Fig. 6. The phosphorylation of p70S6K^Thr389^ (Fig. 5b), rpS6^Ser240/244^ (Fig. 5d), ERK1/2^Thr202/ Tyr204^ (Fig. 5f), AMPKα^Thr172^ (Fig. 5g) showed a time × group interaction effect (*P* < 0.05). There was a main effect of time (*P* < 0.05) for Akt^Ser473^ (Fig. 5a), p70S6K^Thr389^ (Fig. 5b), 4E-BP1^Thr37/46^ (Fig. 5c), rpS6^Ser240/244^ (Fig. 5d), eEF2^Thr56^ (Fig. 5e), ERK1/2^Thr202/Tyr204^ (Fig. 5f), AMPKα^Thr172^ (Fig. 5g). There was a main effect of group (*P* < 0.05) for Akt^Ser473^ (Fig. 5a), p70S6K^Thr389^ (Fig. 5b), rpS6^Ser240/244^ (Fig. 5d), ERK1/2^Thr202/Tyr204^ (Fig. 5f). The phosphorylation of Akt^Ser473^ (Fig. 5a) was increased from baseline at all time points (*P* < 0.05). The phosphorylation of p70S6K^Thr389^ (Fig. 5b) was increased from baseline at 30 min and 60 min in CDX (*P* < 0.05), and it was greater in CDX (83.8-fold) than GLC (18.3-fold) at 60 min (*P* < 0.05). The phosphorylation of 4E-BP1^Thr37/46^ (Fig. 5c) was increased from baseline at 30 min and 60 min (*P* < 0.05 and *P* < 0.0001, respectively). The phosphorylation of rpS6^Ser240/244^ (Fig. 5d) was increased from baseline at all time points in CDX group (*P* < 0.05), and it was greater in CDX than GLC (*P* < 0.05) at 60 min (6.6- vs 16.7-fold) and 180 min (5.6- vs 16.2-fold). The phosphorylation of eEF2^Thr56^ (Fig. 5e) was decreased from baseline at 60 min (*P* < 0.01). The phosphorylation of ERK1/2^Thr202/Tyr204^ (Fig. 5f) was increased from baseline at all time points (*P* < 0.05), and CDX was greater as compared to GLC at all time points (1.1- vs 1.7-fold, 1.2- vs 1.6-fold, 0.9- vs 1.6-fold for 30, 60, 180 min, respectively, *P* < 0.05). The phosphorylation of AMPKα^Thr172^ (Fig. 5g) was greater in CDX than GLC at 180 min (0.9- vs 1.3-fold, *P* < 0.05).

## Discussion

In the present study, we simultaneously assessed orally ingested protein-derived amino acid availability in the circulation using intrinsically labelled meat protein hydrolysate (D_5_-phenylalanine) and myofibrillar protein synthesis of the vastus lateralis muscle after a whole-body resistance exercise in moderately trained younger males. Co-ingestion of CDX with meat protein hydrolysate did not enhance the total availability of protein-derived amino acids in the circulation as determined by the enrichment of serum D_5_-phenylalanine derived from intrinsically labelled meat-protein, serum phenylalanine, EAA, BCAA, and muscle BCAA concentrations compared with GLC. Interestingly, the activation of mTORC1 signaling was higher in CDX than GLC following a whole-body resistance exercise. However, the enhanced activation of mTORC1 signaling did not increase postprandial myofibrillar FSR in CDX as compared to GLC.

Amino acid availability in the circulation is a determinant of muscle protein synthesis [10, 11]. It has been well established that protein/amino acids ingestion stimulates muscle protein synthesis following the recovery from resistance exercise [2, 12, 14, 34–36]. In the previous studies, carbohydrate co-ingestion with protein was hypothesized to augment insulin secretion and enhance muscle protein synthesis following an acute bout of resistance exercise [15–17]. However, we and others previously demonstrated that amino acid availability is decreased when protein is ingested with carbohydrate [15–17] or other macronutrients [18, 19]. The nature of rapid gastric emptying of CDX [27] could be used as an approach to attenuate lower amino acid availability when protein is consumed with GLC. To our knowledge, this is the first study to investigate whether CDX ingestion together with the intake of protein hydrolysate (here meat protein-derived) enhanced the availability of constituent amino acids in the circulation as compared to GLC ingestion. We made use of a previously produced intrinsically labelled meat protein hydrolysate [18] to directly measure exogenous protein-derived amino acid availability (with D_5_-phenylalanine as a tracer) in the present study.

For the main outcome D_5_-phenylalanine enrichment tracer, it appeared in serum from 20 min post-exercise and was maintained until 180 min post-exercise in both CDX and GLC groups **(**Fig. 3a) and iAUC (total availability) of the tracer did not differ between CDX and GLC (Table 3). Further, T_max_ for the tracer was not affected by CDX as compared to GLC. The group means for EAA and BCAA at *t* = − 0:30 and *t* = 0 (Fig. 3) appeared different and were likely to cause the main effect of group in the two-way ANOVA test. Therefore, we further explored if there were any postprandial differences by calculating iAUC from *t* = 0, which revealed no difference for the postprandial rise in EAA and BCAA concentrations between CDX and GLC (Table 3). With these data in mind, the time × group interaction effect for D_5_-phenylalanine enrichment (Fig. 3a, *P* = 0.0072) is hard to explain. Visually, it seems though that the D_5_-phenylalanine enrichment peaks and starts to decrease within the 3-h postprandial period in CDX, whereas it remains high in GLC. However, this is speculative, and more investigations are needed to enlighten this further. Of interest is though, that the T_max_ serum BCAA showed a statistical significance (*P* = 0.049) and T_max_ serum EAA showed a trend towards a statistical significance (*P* = 0.051, Table 3) although this is not the case for D_5_-phenylalanine. However, a flux of different amino acids is regulated by different amino acid transport mechanisms [37], and hence, phenylalanine may not be a representative tracer for the clearance of all amino acids. Overall, we conclude that co-ingestion of CDX with meat protein hydrolysate did not markedly affect serum amino acid availability during the three hours of recovery from a whole-body resistance exercise.

In the present study, insulin was increased above baseline between 30 and 90 min post-exercise drink with no difference between CDX and GLC (Fig. 3d). Previously, studies have shown that CDX intake alone does not affect glucose concentrations as compared to glucose [38] or maltodextrin [39]. Although some amino acids are insulinotropic [40, 41] and a higher insulin secretion is observed when protein is consumed in combination with carbohydrate [15–17] compared to protein alone, the similar serum amino acid concentrations in CDX and GLC groups in this study reject the expectations for the insulin concentrations to be different. However, due to the lack of a “meat protein hydrolysate alone” group in the present study, we cannot conclude whether the co-ingestion of CDX or GLC increased insulin concentration above levels induced by the meat protein hydrolysate alone, although this must be anticipated. The roles of insulin on muscle protein turnover have long been debated. Systematic reviews have concluded that insulin does not have a stimulatory or inhibitory effect on muscle protein synthesis [23], but instead plays an important role in attenuating muscle protein breakdown independent of amino acid availability [42]. The absence of increased muscle BCAA concentrations at 30 and 60 min (where serum concentrations are elevating and peaking, respectively) reveals that the intramuscular disappearance rate of amino acids equals the influx. We did not follow the amino acid tracers further in the intramuscular metabolic pathways, but we suggest that muscle protein synthesis and energy metabolism are responsible for the utilization of excess amino acids. This is due to the concomitant intramuscular availability of glucose, which would meet the major requirement for energy production, dampening the anaplerotic processes. The drop at 180 min in the muscle BCAA concentrations (Fig. 3f) agrees with serum EAA (Fig. 3c), phenylalanine (Fig. 3b), BCAA (Fig. 3e), and insulin concentrations (Fig. 3d) as these substrates had all returned to basal levels. Hence, it is likely that participants were in the postabsorptive period around 180 min post-exercise drink, and that the muscle BCAA pool at that time point was drained by either a net outflux into the circulation or by a request from translation processes or both [43].

It is generally agreed that 20 g of high-quality protein intake is required following resistance exercise (e.g., leg press and knee extension) to maximally stimulate muscle protein synthesis in younger individuals [2, 4]. However, Macnaughton et al. [28] reported findings that may suggest the amount of protein required to stimulate muscle protein synthesis may depend on the amount of muscle recruited during resistance exercise. This is because the demand for exogenous amino acids might be increased when more muscles are used during resistance exercise (whole-body vs unilateral leg) although the amount of lean body mass (LBM) itself does not affect muscle protein synthesis [28]. Macnaughton et al. [28] provided either 0.34 g or 0.68 g protein * LBM^−1^ whey protein in the lower LBM group (59 kg LBM on average), and either 0.26 g protein or 0.52 g protein * LBM^−1^ in the higher LBM group (77 kg LBM on average). In the present study, participants performed a whole-body resistance exercise followed by meat protein hydrolysate (0.6 g * FFM^−1^) intake, which ended up as a mean of 36.3 g of meat protein hydrolysate (range 32.0 to 41.6 g) to our participants, which should provide a stimulus to maximally stimulate myofibrillar FSR following a whole-body resistance exercise. However, as we did not measure the baseline myofibrillar FSR we cannot say whether the myofibrillar FSR was enhanced by an acute bout of whole-body resistance exercise.

Akt/mTORC1 signaling pathway is crucial for muscle protein synthesis and skeletal muscle hypertrophy [7, 44, 45]. Previous studies showed that protein/amino acid feeding [7, 46–48], resistance exercise [35, 49–52], or a combination of both [12, 14, 35, 36, 53] activate Akt/mTORC1 signaling in younger individuals. In line, our whole-body resistance exercise protocol with post-exercise meat protein hydrolysate ingestion increased the phosphorylation of Akt^Ser473^, p70S6K^Thr389^, rpS6^Ser240/244^, 4E-BP1^Thr37/46^, and ERK1/2^Thr202/Tyr204^ and decreased the phosphorylation of eEF2^Thr56^ over the course of 180 min post-exercise period (a main effect for time, *P* < 0.05). Interestingly, the phosphorylation of p70S6K^Thr389^, rpS6^Ser240/244^, and ERK1/2^Thr202/Tyr204^ was greater in CDX compared to GLC during the recovery from a whole-body resistance exercise (a time × group interaction effect, *P* < 0.05), indicating an enhanced translation initiation and elongation in CDX. However, the enhanced mTORC1 signaling did not result in an increased postprandial myofibrillar FSR in CDX (Fig. 4), which is in line with previous studies that demonstrated that co-ingestion of carbohydrate does not further increase FSR compared to protein intake alone [15–17]. The absence of an enhanced postprandial myofibrillar FSR in CDX despite the increased mTORC1 signaling could be explained by no changes of amino acids availability in the circulation as well as muscle BCAA concentrations (Fig. 3). Previous studies have shown that metabolic flux in vivo cannot be predicted by intracellular signaling [54] or mRNA expression level [55]. In support, dissociation between Akt/mTORC1 signaling and muscle protein synthesis in response to amino acids and insulin was previously reported by Greenhaff et al. [56].

The present randomized controlled crossover trial is a robust study design with high statistical power. However, the absence of a meat protein hydrolysate group alone makes it impossible to reveal any effects of CDX or GLC per se, which could have been interesting now that the hypothesized beneficial effects of CDX could not be verified.

## Conclusions

In moderately trained younger males, co-ingestion of CDX with meat protein hydrolysate does not enhance the availability of protein-derived amino acids and myofibrillar FSR as compared to GLC with meat protein hydrolysate during the recovery from a whole-body resistance exercise despite an increased intramuscular signaling.

## Data Availability

The datasets generated during and analyzed during the current study are available from the corresponding author on reasonable request.

## Abbreviations

mTORC1: Mammalian target of rapamycin complex 1
CDX: Cluster dextrin
iAUC: Incremental area under the curve
T_max_: Time to reach maximum concentration
DXA: Dual X-ray absorptiometry
FFM: Fat-free mass
BMI: Body mass index
RM: Repetition maximum
GLC: Glucose
PTC: Phenylthiocarbamyl
LC-MS/MS: Liquid chromatography tandem mass spectrometer
ELISA: Enzyme-linked immunosorbent assay
NAP: *N*-Acetyl-propyl
GC-C-IRMS: Gas chromatography combustion isotope ratio mass spectrometry
p70S6K: 70 KDa S6 protein kinase
4E-BP1: Eukaryotic initiation factor 4E-binding protein
Akt: Protein kinase B
rps6: Ribosomal protein S6
eEF2: Eukaryotic elongation factor 2
FSR: Fractional synthetic rate
BCAA: Branched-chain amino acids
EAA: Essential amino acid
TTR: Tracer to tracee ratio
MPE: Mole percent excess
IU: International unit
